# Preemptive Analgesic Efficacy of Dexamethasone and Diclofenac in Mitigating Post-surgical Complications After Mandibular Third-Molar Surgery: A Systematic Review

**DOI:** 10.7759/cureus.42709

**Published:** 2023-07-30

**Authors:** Dr.Yashwanth V Satyanarayana Killampalli, Monal Yuwanati, Murugesan Krishnan, Santhosh P Kumar, Melvin George, Saravanan Lakshmanan

**Affiliations:** 1 Oral and Maxillofacial Surgery, Saveetha Dental College and Hospitals, Saveetha Institute of Medical and Technical Sciences, Saveetha University, Chennai, IND; 2 Oral Pathology, Saveetha Dental College and Hospitals, Saveetha Institute of Medical and Technical Sciences, Saveetha University, Chennai, IND

**Keywords:** impacted third molars, diclofenac sodium, dexamethasone, pre-emptive analgesia, third molar surgery

## Abstract

Mandibular third-molar extraction is a frequently executed minor oral surgical procedure, with a subsequent recovery period lasting several days. Typically, preemptive administration of non-steroid anti-inflammatory drugs (NSAIDs) and steroids has been employed, resulting in a notable decrease in postoperative complications like pain, facial swelling, trismus, and alveolar osteitis. This systematic review's primary goal was to investigate the efficacy of preemptive analgesia with dexamethasone and diclofenac in minimizing the post-surgical complications following the surgical extraction of the mandibular third molars. The systematic search was carried out to identify relevant literature in digital databases including PubMed®, Cochrane Library, Web of Science, and Scopus, from January 1990 to January 2022. The search used specific keywords. The randomized clinical trials assessing the efficacy of dexamethasone and diclofenac or dexamethasone alone compared to diclofenac or placebo as preemptive analgesics were considered inclusion criteria for this systematic review. Case reports, literature reviews, letters to the editor, and non-English publications were not included. Two authors screened the titles and abstracts, and articles fulfilling the study criteria were included. After reading the full text and data collection, analysis was performed. The included article's bias was evaluated by the Risk of Bias 2 (RoB 2) tool. A digital database search yielded a total of 207 articles. After excluding duplicates and articles written in languages other than English, 90 were removed. Based on the title and abstract, out of 177, 95 studies were excluded. After full-text reading of 22 articles, 17 were eliminated because they did not meet the inclusion and exclusion criteria. The remaining five studies were found eligible and included in the systematic review. Four studies were of low risk, while one study had some concerns. Two studies evaluated the combination of dexamethasone with diclofenac, while three evaluated dexamethasone alone. Total samples included samples of 436 third-molar surgeries in 420 patients. There was a substantial decrease in the mean pain score and swelling measurement when diclofenac alone was compared with coadministration of diclofenac and dexamethasone. Preemptive administration of dexamethasone and diclofenac has been shown to effectively reduce pain and facial swelling, with the exception of trismus, in third-molar surgeries when compared to diclofenac alone. As a result, it is recommended to administer these drugs prior to the commencement of third-molar extraction. However, further research is mandatory, specifically good quality randomized controlled trials involving large cohorts, in order to assess any significant variations and validate these findings.

## Introduction and background

Mandibular third-molar surgery's most commonly faced problem is moderate-to-severe acute postoperative sequelae such as pain, swelling, and reduced mouth opening or trismus. Many clinicians tried to reduce these complications with routinely used non-steroid anti-inflammatory drugs (NSAIDs), however with limited success. This necessitates an alternate approach to managing the previously mentioned complications. In dentistry and medicine, NSAIDs have made a major impact on the management of postoperative pain. Prior to surgical trauma, NSAIDs have well-established mechanisms for their efficacy. The first advantage may be pharmacokinetic in nature. With the preemptive administration of NSAIDs prior to the onset of pain, drug absorption would have begun and therapeutic blood levels would have been reached prior to the onset of pain. Second, the presence of a cyclooxygenase inhibitor at the surgical site could decrease the secretion of prostaglandins and prostacyclins, which are linked to hyperalgesia and edema. Corticosteroids like dexamethasone and betamethasone are another preventive measure for minimizing postoperative edema and trismus after third-molar extractions. Postoperative edema may be caused to some extent by the conversion of phospholipids to arachidonic acid by phospholipase A2 and the subsequent production of inflammatory mediators including leukotrienes, prostacyclins, prostaglandins, and thromboxanes A2. Steroid use may hinder the initiation of this process. Schultze-Mosgau et al. researched the combined use of ibuprofen and methylprednisolone for pain alleviation and found this combination possesses effective analgesic and anti-inflammatory properties [1]. He reported that a single dose of a glucocorticoid suppresses circulating levels of cortisol and beta-endorphin and lowers tissue levels of bradykinin. As known, bradykinin and kallidin are the two kinins that act both separately and synergistically with the arachidonic acid pathway to produce both hyperalgesia and increased vascular permeability. 

Additionally, oral surgery clinical trials have proven the hypothesis that preemptive NSAIDs and corticosteroids prove beneficial in deferring and preventing numerous postoperative complications. The interactions between the mechanisms of action of NSAIDs and steroids suggest that combination therapy could offer anti-inflammatory and pain relief without adverse effects. However, the evidence in the literature regarding their efficacy is limited. Thus, it is important to find evidence of the effectiveness of the combination of dexamethasone and diclofenac or dexamethasone alone in reducing postoperative complications of third-molar surgery.

## Review

The study protocol and research question

This systematic review was reported according to Preferred Reporting Items for Systematic Reviews and Meta-Analyses (PRISMA) checklist [2]. The research question of the systematic review was "Is preemptive analgesia with dexamethasone and diclofenac effective in lowering postoperative discomfort like pain, swelling, and trismus following mandibular third molar surgery." The population, intervention, control, and outcome (PICO) elements of the systematic review are shown in Table 1.

**Table 1:** PICO elements of the systematic review PICO, population, intervention, control, and outcome

Elements	PICO
Population	Patients undergoing mandibular third molar surgery, impaction, wisdom tooth surgery
Intervention	Dexamethasone and diclofenac, dexamethasone
Comparison	Placebo, without any analgesics, diclofenac
Outcome	Postoperative control of pain, swelling, trismus

The comprehensive search was carried out in digital databases such as PubMed, Web of Science, Scopus, and Cochrane Library until January 2022. The selection criteria included controlled clinical trials and randomized clinical trials utilizing dexamethasone with or without diclofenac, comparing them to diclofenac placebo as preemptive analgesics. No age, gender, or demographic restrictions were applied. However, studies published in languages other than English were excluded. The inclusion and exclusion criteria for the study are provided in Table 2.

**Table 2:** Inclusion and exclusion criteria NSAIDs, non-steroid anti-inflammatory drugs

Inclusion	Exclusion
Study types: randomized controlled trials and clinical trials	Study types: case reports/case series
Publications in the English language	Publications other than the English language
Participants undergoing surgical extraction of mandibular third molar	Third molar extraction along with pathologies
Intervention: diclofenac and dexamethasone as a combination or alone as preemptive analgesia in lower third molar surgery	Studies in which other NSAIDs combination with any other steroids or drugs were used
Comparison: placebo, without any analgesics, any other analgesics as preemptive analgesia	
Outcome measures: postoperative pain, swelling, trismus	

Search Strategy and Sources

The search strategy was prepared using PICO, and databases (PubMed, Cochrane Database of Systematic Review, Scopus, and Web of Science) were searched. The search terms included "randomized controlled trial," "controlled clinical trial," "third molar surgery," "mandibular third molar impaction," "dexamethasone," "diclofenac," "preemptive," "pain," "swelling," "trismus," and "postoperative complications." In addition, the British Journal of Oral and Maxillofacial Surgery, International Journal of Oral and Maxillofacial Surgery, Journal of Oral and Maxillofacial Surgery, Journal of Cranio Maxillofacial Surgery, and Quintessence International Journals were searched for additional relevant papers. Further cross-references were manually searched.

**Figure 1:**
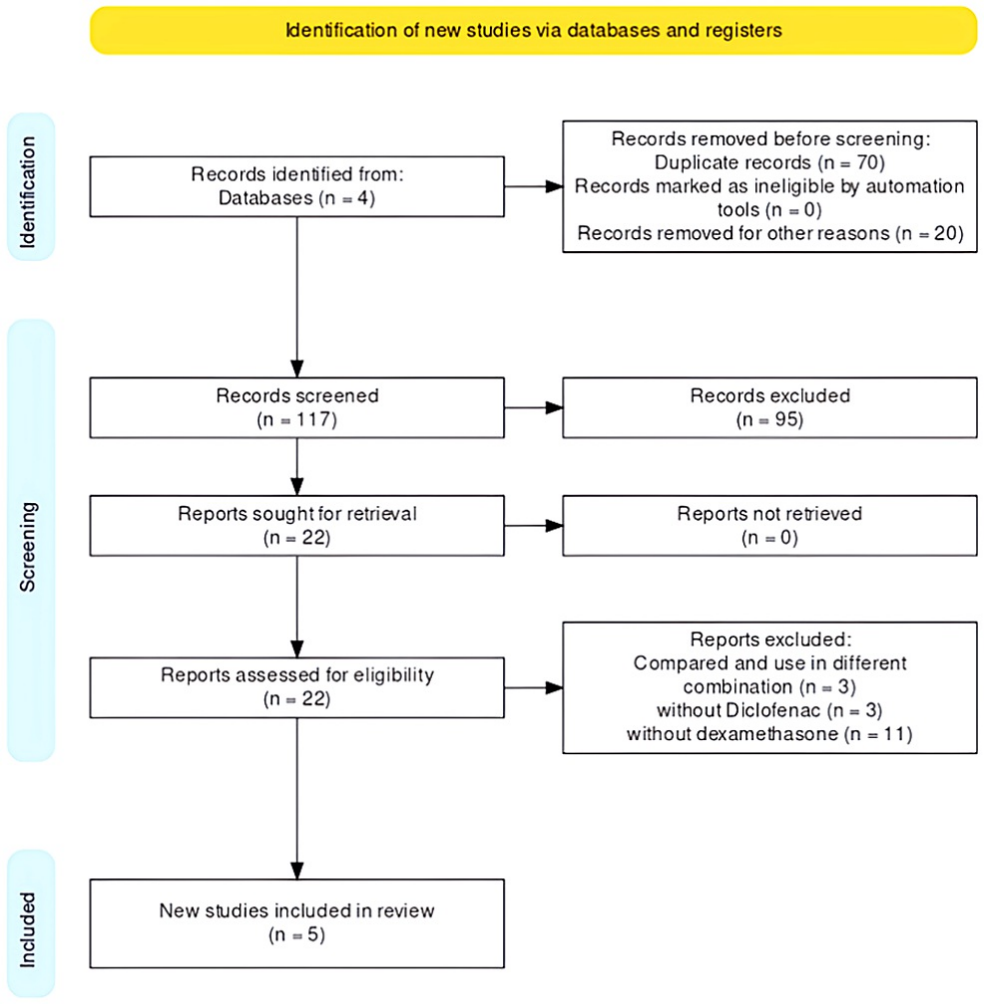
PRISMA flowchart PRISMA, preferred reporting items for systematic reviews and meta-analyses

Two authors independently searched the included databases. Duplicated studies across databases were removed. Furthermore, two authors independently screened titles to identify relevant studies based on predetermined inclusion and exclusion criteria. Any discrepancies between the authors were resolved through discussion. Abstracts were evaluated when title information was insufficient. The final study inclusion was determined independently by both authors after full-text reading. Additional studies were identified through scrutiny of reference lists from full-text articles. After finalizing the inclusion of the study in the systematic review, data was extracted. The extracted data included author, journal, year, study design, sample size, participants and groups, methodology, outcome assessment, and results and conclusions. Figure 1 depicted the search strategy according to the PRISMA guidelines. The outcome of interest in the systematic review included evaluating post-surgical pain, facial swelling, and reduction in mouth opening using various measurements and scales.

Risk of Bias and Quality Assessment

The risk of bias for each study was evaluated independently by the review authors, and discrepancies regarding the risk of bias were resolved through discussion. Quality assessment was done using the Cochrane RoB (Risk of Bias) 2 tool [3].

Summary Synthesis

The descriptive analysis was carried out. The results were summarized by grouping the information. The results are categorized into outcomes, i.e., pain, swelling, and trismus. Furthermore, descriptive analyses were performed for the type of drug, dose, and time of outcome analysis.

Results

Study Selection

The MeSH (Medical Subject Headings) terms/keywords were used to conduct a digital search in the four search engines: PubMed®, Cochrane Library, Web Of Science, and Scopus, which obtained a total of 207 articles. Seventy articles were excluded due to duplication and 20 articles were removed due to other languages. The remaining article abstracts were reviewed independently. After reading titles and abstracts, 95 articles were excluded; among them 22 articles remained, and 17 were excluded after reading the full text [4-20]. Five articles remained for the final assessment. 

Study Characteristics

Table 3 and Table 4 contain a detailed description of the included studies' characteristics and an evaluation of their outcomes using clinical parameters. Five included studies were randomized clinical trials conducted in Nigeria and Brazil. Total samples included 436 third molar surgeries in 420 patients. The age of the patients was between 16 years and above. A total of 219 males and 201 females were included. Three patients were excluded. Two studies were double-blinded studies, one study was a single-blinded study, and one study was split mouth triple-blinded study. and another one is an open-label pilot study. With the exception of the study conducted by Simone et al., all other studies assessed pain, swelling, and mouth opening as outcome measures. However, Simone et al. focused solely on evaluating pain and the total amount of rescue medications used [21].

**Table 3:** Characteristics of included studies RCT, randomized control trial; Dexa, dexamethasone; Diclo, diclofenac sodium; Para, paracetamol

Author, year	Journal, country	Type of study	Sample size, Age	Groups and drug usage, form	
Babatund Olamide Bamgbose et al., 2005 [22]	Biomed Central Journal, Nigeria	Prospective randomized double-blind study	Sample size: 100; Age: 16 years and above; 52 men and 48 female	2 Groups	Group 1: Dexa 8 mg IV and Diclo 50 mg, Oral
					Group 2: Diclo 50 mg, Oral
Babatunde Olamide Bamgbose et al., 2006 [23]	Current Therapeutic Research, Nigeria	Prospective, randomized, open-label pilot study	Sample size: 150, age: 18 and 45 years; 75 men and 76 female	3 Groups	Group 1: Dexa 8 mg and Diclo 50 mg, Oral
					Group 2: Dexa 8 mg and Para 1 g, Oral
					Group 3- Diclo 50 mg, Oral
José Leonardo Simone et al., 2013 [21]	Brazilian Oral Research, Brazil	Randomized, double-blind, parallel, placebo-controlled study	Sample size: 54, age: 16-28 years; 19 men and 35 female	3 Groups	Group 1- Diclo 50 mg, Oral
						Group 2- Dexa 8 mg, Oral
						Group 3: Placebo
						Thiago César Lima et al., 2017 [24]	Journal of Oral and Maxillofacial Surgery, Brazil	Split-mouth, RCT, triple-blind study	Sample size: 30, age: 18-35 years; 3 male and 12 female	2 Groups	Group 1: Dexa 8 mg, Oral
											Group 2: Diclo 50 mg with codeine 50 mg, Oral
						Rakesh B. Nair et al., 2013 [25]	The Journal of Contemporary Dental Practice	Randomized controlled clinical trial	Sample size: 100; Age: 18 and above	2 Groups	Group 1: Dexa 4 mg, sub-mucosal
					Group 2: control group, no drug					

**Table 4:** Characteristics of included studies CRS, category rating scale; VAS, visual analog scale

Study	Method of evaluation	Mean values		Outcomes
Babatund Olamide Bamgbose et al., 2005 [22]	Pain: 4-point Likert type pointer CRS (mean±standard deviation)	Group 1: day 1, 0.62±0.6		Dexamethasone and diclofenac combination showed notable differences in administration, excluding trismus relief. Results were statistically significant in pain and swelling.
		Group 1: day 2, 0.5±0.5		
		Group 2: Day 1, 1.64±0.9		
		Group 2: Day 2, 1.3±0.62		
	Facial swelling in millimeters: using a tape (mean±standard deviation)	Group 1: Day 1, 30.9±1.6		
		Group 1: Day 2, 31.0±1.58		
		Group 2: Day 1, 31.7±1.6		
		Group 2: Day 2, 32.04 0±1.5		
	Mouth-opening in millimeters: Using vernier caliper (mean±standard deviation)	Group 1: Day 1, 36.0±11.2		
		Group 1: Day 2, 38.1±10.05		
		Group 2: Day 1, 39.2±11.3		
		Group 2: Day 2, 36.0±10.02		
Babatunde Olamide Bamgbose et al., 2006 [23]	Pain: 4-point Likert type pointer CRS in percentage, Day 1	Group 1; None, 22 (44)		Dexamethasone and diclofenac K reduced pain and facial swelling significantly compared to the other groups. Trismus relief was similar.
		Group 1: Mild, 25 (50)		
		Group 1: Moderate, 3(6)		
		Group 1: Severe, 0		
		Group 2: None, 11 (22)		
		Group 2: Mild, 32 (64)		
		Group 2: Moderate, 7 (14)		
		Group 2: Severe, 23(46)		
		Group 3: None, 12 (24)		
		Group 3: Mild, 23 (46)		
		Group 3: Moderate, 11 (22)		
		Group 3: Severe, 4 (8)		
	Facial swelling: a tape in (mean±standard deviation)	Day 1: Group 1, 30.9 (1.6)		
		Day 1: Group 2, 30.9 (1.5)		
		Day 1: Group 3, 31.7 (1.6)		
		Day 2: Group 1, 31.0 (1.6)		
		Day 2: Group 2, 31.2 (1.5)		
		Day 2: Group 3, 32.0 (1.5)		
	Mouth opening: vernier caliper in millimeters (mean±standard deviation)	Day 1: Group 1, 36.0 (11.2)		
		Day 1: Group 2, 38.8 (12.6)		
		Day 1: Group 3, 39.2 (11.3)		
		Day 2: Group 1, 38.1 (10.1)		
		Day 2: Group 2, 38.1 (10.8)		
		Day 2: Group 3, 36.0 (10.0)		
José Leonardo Simone et al. [21]	Pain: VAS (mean±standard deviation, pain scores in the entire period)	Dexa, 1.9±0.4		The dexamethasone group had lower pain intensity compared to diclofenac and placebo. Not much difference in total rescue medication among them.
		Placebo, 3.0±0.5		
		Diclo, 2.7±0.7		
	The total amount of rescue medications of paracetamol used (mean±standard deviation)	Dexa, 2.1±3.4		
		Placebo, 3.0±4.0		
		Diclo, 1.7±2.7		
Thiago César Lima et al. [24]	Pain: 10-point numerical rating scale (mean±standard deviation)	24 hr	Group 1, 3.2±2.1	Dexamethasone provided better pain control and reduced swelling compared to sodium diclofenac with codeine. No difference in mouth opening and rescue analgesics between groups.
			Group 2, 3.9±2.2	
		48 hr	Group 1, 3.4±2.0	
			Group 2, 5.2±2.1	
		72 hr	Group 1, 3.1±2.6	
			Group 2, 4.0±2.4	
	Swelling: using a tape measure by 3 pointer scale in millimeters (mean±standard deviation)	24 hr	Group 1, 0.52±0.3	
			Group 2, 0.11±0.7	
		48 hr	Group 1, 0.77±0.5	
			Group 2, 1.45±0.7	
		72 hr	Group 1, 0.60±0.4	
			Group 2, 1.12±0.7	
		7 days	Group1, 0.18±0.2	
			Group 2, 0.28±0.3	
	Reduction in mouth opening in millimeters (mean±standard deviation)	24 hr	Group 1, 16.6±9.9	
			Group 2, 17.3±6.9	
		48 hr	Group 1, 16.6±10.1	
			Group 2, 19.7±6.5	
		72 hr	Group 1, 15.1±10.0	
			Group 2, 17.2±7.1	
		7 days	Group 1, 9.11±8.8	
			Group 2, 10.0±7.0	
Rakesh B. Nair et al. [25]	Pain: 9-point VAS (mean±standard deviation)	2nd postoperative day	Group 1, 3.1±1.70	Submucosal dexamethasone reduces pain and facial swelling more than the control group but no difference in reduction in mouth opening
			Group 2, 3.73±1.56	
		7th postoperative day	Group 1, 1.27±1.11	
			Group 2, 1.98±1.49	
	Swelling: 3-pointer measurements scale (mean±standard deviation)	Group 1: 2nd postoperative day	A point, 111.02±5.7	
			B point, 133.57±10.3	
		Group 2: 2nd postoperative day	A point, 115±6.6	
			B point, 137.9±10.5	
		Group 1: 7th postoperative day	A point, 110.8±5.7	
			B point, 130.8±20.0	
		Group 2: 7th postoperative day	A point, 113.6±6.2	
			B point, 135.7±10.3	
	Reduction in mouth opening (mean±standard deviation)	Preoperative	Group 1, 42.27±6.7	
			Group 2, 43.12±6.7	
		2nd postoperative day	Group 1, 3.73±1.5	
			Group 2, 3.1±1.7	
		7th postoperative day	Group 1, 1.27±1.1	
			Group 2, 1.98±1.4	

Pain Assessment

Evaluation of pain was done using a four-point Likert scale in two trials conducted by Bamgbose et al. [22,23,26]. In these two studies, postoperative pain assessment was performed on Day 1 and Day 2. However, in the 2005 study the postoperative pain was measured as a mean score whereas in the 2006 study, the postoperative pain was measured as a percentage. Both studies concluded that the combination of dexamethasone with diclofenac exhibited a significantly greater relief of postoperative pain compared to diclofenac alone. Simon et al. utilized a visual analog scale (VAS) to record postoperative pain [21]. Their study concluded that the dexamethasone group exhibited lower postoperative pain intensity compared to both the diclofenac and placebo groups. Lima et al. and Rakesh B Nair et al. implemented a 10-point numerical rating scale to assess postoperative pain over a period of three days [24,25]. Lima et al. concluded that dexamethasone provided superior postoperative pain control compared to diclofenac [24]. Although, Rakesh B. Nair et al. found that dexamethasone alone demonstrated a reduction in postoperative pain over the control group [25].

Swelling Assessment

Assessment of facial swelling was done by a tape measure kept from the tragus-gonion to the other side tragus in two studies conducted by Bamgbose et al. [22,23]. In both studies, the assessment was conducted on Day 1 and Day 2 to measure postoperative swelling, recorded as the mean score±standard deviation. Both studies concluded that the combination of dexamethasone with diclofenac exhibited a significantly greater reduction of postoperative swelling compared to diclofenac alone. Lima et al. and Rakesh B Nair et al. utilized a tape measure and a three-point measurement scale to evaluate facial swelling over a period of seven days [24,25]. Lima et al. concluded that dexamethasone provided superior control of swelling compared to diclofenac [24]. Although, Rakesh B. Nair et al. concluded that dexamethasone demonstrated a reduction in swelling over the control group [25].

Trismus Assessment

Trismus, or restricted mouth-opening, was evaluated by measuring interincisal distance in all included studies. Two studies conducted by Bamgbose et al. used a vernier calibrated caliper whereas Lima et al. and Rakesh B. Nair et al. used a simple caliper [8,22-24]. In Bamgbose et al. studies, the assessment was performed on Day 1 and Day 2 where postoperative trismus (mouth opening) was recorded as the mean±standard deviation [22,23]. Both studies concluded that the relief of trismus was comparable across all the groups. Similar findings were reported by Lima et al. and Rakesh B. Nair et al [24,25].

Risk of Bias

**Figure 2:**
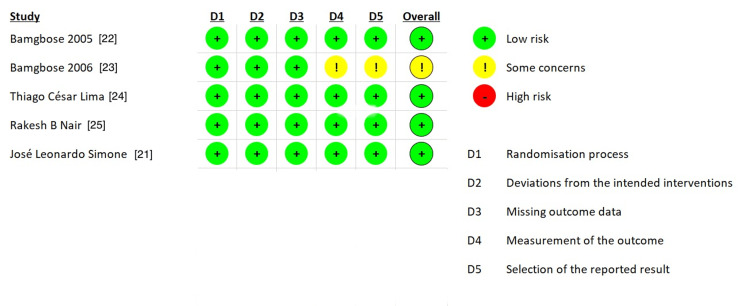
Risk of bias assessment of included articles

After quality assessment using the RoB 2 tool, a risk of bias for five studies was found. Out of five, four were of low risk; however, one was having some concerns (Figure 2). This concern was mainly due to a lack of open-label clinical trials.

Level of Evidence

The level of evidence for included studies was assessed by the Oxford Centre for evidence-based medicine. Included studies were randomized controlled trials, hence the level of evidence was Ib.

Discussion

Third molar surgeries are routine procedures performed in dental clinics with minimal complications. However, in certain cases, postoperative complications such as pain, swelling, trismus, and infection can occur. While these complications are typically short-term and resolve over time, they can become more severe and prolonged, causing discomfort and negatively impacting a patient's quality of life. Several factors contribute to the development of these symptoms, including patient demographics, oral hygiene, medication, compliance with postoperative instructions, presence of preexisting dental conditions, and surgical factors such as duration, complexity, and magnitude. Despite these factors, some patients continue to experience symptoms that require extended postoperative medication. Without appropriate medication, the severity of symptoms can worsen, leading to prolonged recovery times. Clinicians commonly prescribe analgesics to alleviate symptoms and promote postoperative recovery. However, the conventional practice of prescribing these drugs after surgery has not demonstrated significant advantages in reducing postoperative pain and recovery time.

To address this, clinicians have started implementing preemptive administration of analgesics. Ong et al. and Pektas OZ et al. examined preemptive analgesia using tramadol with ketorolac and diflunisal with lornoxicam, respectively, in the context of third molar extraction [27,28]. Both studies showed no statistically significant differences between the groups in terms of postoperative rescue analgesics intake and postop pain values. However, subsequent research revealed that diclofenac and dexamethasone were more effective in the reduction of pain and swelling, excluding trismus, as demonstrated by Bamgboos et al. [22,23]. A systematic review was conducted to assess the efficacy of preemptive analgesics, specifically diclofenac and dexamethasone. The review revealed that the combination of these medications was superior in reducing postoperative pain and swelling compared to diclofenac alone. These findings contradicted a study by Joshi et al., which found no significant difference in postoperative dental pain among patients receiving preoperative ibuprofen, diclofenac, paracetamol with codeine, or placebo tablets. Similarly, Hyrkas et al. reported no significant difference in analgesic efficacy with preoperative oral administration of diclofenac sodium following third molar surgery [29]. However, a study by Bamgboos et al. supported the use of preemptive analgesia to prevent the amplification of pain after surgery [22,23].

The coadministration of corticosteroids with NSAIDs has been proposed as an effective technique to reduce postoperative inflammation. Cortisol and synthetic cortisol analogs can interfere with inflammatory processes, thereby suppressing characteristic symptoms. NSAIDs act by regulating prostaglandin synthesis, which is closely associated with pain, inflammation, and fever. By inhibiting the cyclooxygenase enzyme system, NSAIDs effectively reduce prostaglandin production. Administering NSAIDs prior to surgery has shown particular effectiveness in alleviating postoperative pain. Another study by de Sousa Santos JA involving third molar surgery patients demonstrated reduced pain and swelling after taking a preemptive combination of medications, suggesting a synergistic effect [18]. The systematic review included pain outcomes assessed across five studies using three different evaluation scales. Irrespective of pain scales used for evaluations, all included studies reported a reduction in postoperative pain after preemptive administration of drugs either in combination or alone. One study by Lima et al. that used dexamethasone compared to diclofenac in pain reduction found that dexamethasone is better than diclofenac [24]. However, when dexamethasone and diclofenac were coadministered, the pain reduction was more compared to diclofenac alone. This could be because of the synergistic effect of both drugs which need to be studied in the future.

Facial swelling is another complication usually observed after third molar surgery due to inflammation and edema. The facial swelling usually involves the buccal and preauricular region, which often causes discomfort due to the involvement of inflammation of the muscle and mucoperiosteal flap. Therefore, it is important to have less inflammation to reduce facial swelling and discomfort. In the present systematic review, all studies except Simon et al. measured facial swelling after preemptive analgesia where dexamethasone and diclofenac were effective in reducing facial swelling [21]. This could be attributed to the anti-inflammatory properties of diclofenac. In one study by Lima et al., when dexamethasone and diclofenac were compared alone, it was found that dexamethasone was more effective suggesting that dexamethasone might have exerted an anti-inflammatory effect through suppression of inflammatory response [24]. However, dexamethasone should be used cautiously. 

In third molar surgery, patients may have trismus due to mild injury medial pterygoid muscle during the inferior alveolar nerve block. Trismus usually causes a reduction in mouth opening and may persist for a longer duration affecting day-to-day activities like mastication and chewing. It is suggestive that preemptive medication may assist in reducing the trismus. However, the present systematic review did not find any evidence regarding the reduction of trismus irrespective of preemptive analgesia using a combination or single NSAIDs or steroids. The exact reason for this observation could not be ascertained. However, the development of trismus involves multiple factors such as acute inflammation, stripping of the lowest part of the temporalis tendon during flap elevation, traction of buccal flaps, spasmodic contraction of the medial pterygoid, multiple pricks/traumas during infiltration, which could explain this observation [30].

It is important to acknowledge certain limitations of this study. The included trials exhibited heterogeneity in terms of outcome evaluation, different time points, dosage, and mode of administration. Additionally, the sample size involved in these studies was relatively small, which limited our ability to identify meaningful differences. Furthermore, the use of different pain evaluation scales, such as the standard VAS, and Likert-type pointer category rating scale restricted our ability to conduct a meta-analysis.

## Conclusions

Preemptive administration of dexamethasone and diclofenac has been shown to effectively reduce pain and facial swelling, with the exception of trismus, in third molar surgeries when compared to diclofenac alone. As a result, it is recommended to administer these drugs prior to the commencement of third molar extraction. However, it is important to note that further research is needed, specifically good quality randomized controlled trials involving large cohorts, in order to assess any significant variations and validate these findings.
